# Editorial: Proceedings of iMMM 2019 – International Molecular Mycorrhiza Meeting

**DOI:** 10.3389/fpls.2020.627988

**Published:** 2020-12-18

**Authors:** Paola Bonfante, Luisa Lanfranco, Alessandra Salvioli di Fossalunga, Stefano Ghignone, Veronica Volpe, Valentina Fiorilli, Silvia Perotto, Raffaella Balestrini, Andrea Genre

**Affiliations:** ^1^Department of Life Science and Systems Biology, University of Turin, Turin, Italy; ^2^Institute for Sustainable Plant Protection, Italian National Research Council, Turin, Italy

**Keywords:** plant symbiosis, mycorrhiza, endomycorrhiza, ectomycorrhiza, endophytes

This Research Topic was launched on the occasion of the 4^th^ International Molecular Mycorrhiza Meeting (IMMM 2019), held in Torino, Italy, on 7–8^th^ February 2019 (Figure 1) and has collected 13 selected contributions from the IMMM 2019 meeting attendants.

**Figure 1:**
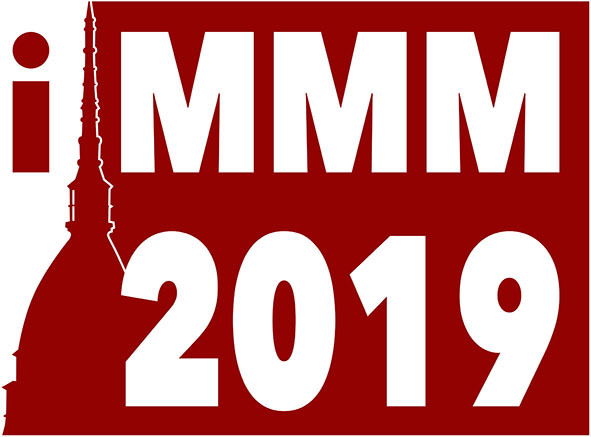
Logo of the iMMM 2019 conference.

The articles include original research, methods, reviews, and perspective papers covering most of the main topics presented at the meeting. These embrace molecular, cellular and nutritional aspects of mycorrhizas, the beneficial plant associations with soil fungi, as well as methodological approaches. Mycorrhizal fungi are considered key components of natural and agricultural ecosystems because they colonize the roots of most land plants developing different types of symbiotic interactions that greatly contribute to plant growth and health. A broad interest is focused on mycorrhizal symbioses, in particular arbuscular mycorrhiza (AM), for their potential contribution to agricultural practices that reduce the use of chemical fertilizers and pesticides and support sustainable crop production to feed a growing human population in a scenario of global climate change. It is therefore not surprising that the majority of contributions to both the meeting and the Research Topic concerned the AM symbiosis. In addition to agricultural applications, the great interest for AM symbiosis also derives from its ecological success (being found in over 72% of land plant species), its long evolutionary history (since plants and AM fungi started to live together at least 400 million years ago), and the refined coordination between major developmental and physiological processes of both partners (Genre et al., 2020).

In line with the meeting's major focus, many studies illustrate molecular aspects of mycorrhizal interactions. On the fungal side, Chen et al. propose new evidence in support of nuclear recombination in dikaryotic AM fungal isolates, reinforcing the hypothesis of the occurrence of parasexual or sexual reproduction processes and adding to the lively debate on this topic in the scientific community (Reinhardt et al., 2020). The paper by Miranda Hart's group offers food for thought, focusing on the unexplored effects of environmental constraints on AM fungal genomes. The article (Kokkoris and Hart) discusses whether the use of transformed root cultures—which is very common for *in vitro* mass production of AM fungi—may generate domesticated AM fungal strains, genetically and functionally different from their wild relatives. By deep mining the *Rhizophagus irregularis* genome, Gómez-Gallego et al. identified two fungal genes encoding Cu transporters of the copper transport (CTR) family and two alternative spliced variants of a third gene possibly involved in Cu perception and tolerance.

A second group of contributions investigates plant molecular responses to AM symbiosis in combination with biotic and abiotic stress. Balestrini et al. report changes in the transcriptome of mycorrhizal tomato roots in response to drought and root-knot nematode infection (two major causes of tomato yield losses) and highlight how plant and fungal gene regulation acts synergistically to face adverse environmental condition. Novel results on the role of AM fungi in plant nutrition are provided by Calabrese et al. Their analysis of the extra- and intra-radical mycelium transportome in *R. irregularis* highlights the role of the common mycorrhizal network in connecting perennial/C3 and annual/C4 hosts and in distributing mineral and organic nutrients between partners.

The role of AM fungi in plant nitrogen nutrition has been demonstrated in several *in vitro* studies, but Thirkell et al. now extend the investigation to field conditions, demonstrating a nitrogen acquisition from soil cores that are not reached by the root system, and providing valuable information for agricultural management practices.

The beneficial role of AM symbiosis in plant nutrition is also discussed in the review by Xie et al., who propose that a better understanding of the cross-talk between phosphate, zinc, and iron homeostasis and signaling in mycorrhizal crops can lead to new strategies for nutrient management. A related topic is discussed by Stuart and Plett in the field of ectomycorrhizas (ECM). Their study illustrates the impact of environmental conditions on nitrogen acquisition and transport by ECM fungi, stressing the importance of extending molecular studies of ECM to provide more solid bases to understand their complex ecology and multiple interactions.

Concerning the cellular aspects of plant-fungal interactions, two contributions investigate plant membrane dynamics in AM development through cell and molecular biology approaches. Russo et al. show the involvement of endocytosis-based processes in perifungal membrane remodeling during the development of the symbiotic interface compartment in legume and non-legume hosts, while Huisman et al. provide a deep insight in the characterization of *M. truncatula* SNARE proteins that have a specific role in symbiosis-associated exocytic mechanisms.

Another acknowledged advantage of AM plants is their improved resistance to pathogens, through the basal activation of mild defense mechanisms. However, investigations on how pathogen resistance may also modulate plant responsiveness to mycorrhization are limited. Hilbert et al. analyze root colonization by the beneficial endophyte *Serendipita indica* and by the AM fungus *Funneliformis mosseae* in a pathogen-resistant barley mutant. Their results demonstrate that while root colonization by the beneficial endophyte is reduced, AM development is promoted in the barley mutant, confirming the uniqueness of the plant-fungus relationship in AM interactions.

Lastly, two studies propose new experimental methods to investigate AM interactions. Das et al. describe a hydroponic set-up to obtain AM colonized roots under strictly controlled conditions suitable for testing specific nutrient concentrations or candidate signaling molecules, while Chiapello et al. propose “Ramf,” an open-source R package for the elaboration of quantitative data of AM colonization to rapidly obtain statistical summaries and publication-ready plots.

Less than 2 years after the acceptance of the first manuscript, the IMMM 2019 Research Topic has gained more than 40,000 views and 6,000 downloads. This success mirrors the growing audience of IMMM meetings and the increasing interest in mycorrhizal interactions both for their applicative potential and their uniqueness as biological models for elucidating inter-kingdom relationships.

The 5^th^ IMMM conference was planned to take place in Shanghai, China in July 2020. Unfortunately, the SARS-CoV-2 outbreak has imposed a rescheduling of the meeting, but we are looking very much forward to welcoming an even larger audience and more outstanding research at the next edition. For more information and updates, please visit the IMMM 2019 website at http://www.societabotanicaitaliana.it/immm2019/.

## Author Contributions

All authors listed have made a substantial, direct and intellectual contribution to the work, and approved it for publication.

## Conflict of Interest

The authors declare that the research was conducted in the absence of any commercial or financial relationships that could be construed as a potential conflict of interest.
